# Supplementary material for: Direct Writing Corrugated PVC Gel Artificial Muscle via Multi-Material Printing Processes
Source: Polymers (Basel). 2021 Aug 15;13(16):2734. doi: 10.3390/polym13162734 (PMC8400140; doi:10.3390/polym13162734)
Supplement: Supplementary file 1 [file polymers-13-02734-s001.zip › Support materials.pdf]

Supporting Information

In this paper, the 3D printing used a multi-nozzle direct writing device (Figure S1, Supporting Information1). The device included a computer control system, a 3D positioning stage, an ink extrusion system, and a heating platform. The composition of 3D printing system is shown in figure S2. According to the input G codes, PC calculates the motion coordinates and other parameters, and controls the rotational speed of the stepping motor by controlling the command pulse frequency input to the stepping motor, and controls the rotation angle of the stepping motor by controlling the number of command pulses. At the same time, the lead screw nut structure in the 3D positioning stage converts the rotary motion of the stepping motor into the linear motion of the nozzle. In this way, PC can control the three-dimensional movement of the nozzle by controlling the movement of the three stepping motors through the above method. Multiple nozzles cannot work at the same time, and only one material can be printed at the same time. The heating platform is to control the working temperature of the printing table. In other work, the printing performance of some specific materials may need to be controlled by controlling the working temperature of the printing table.

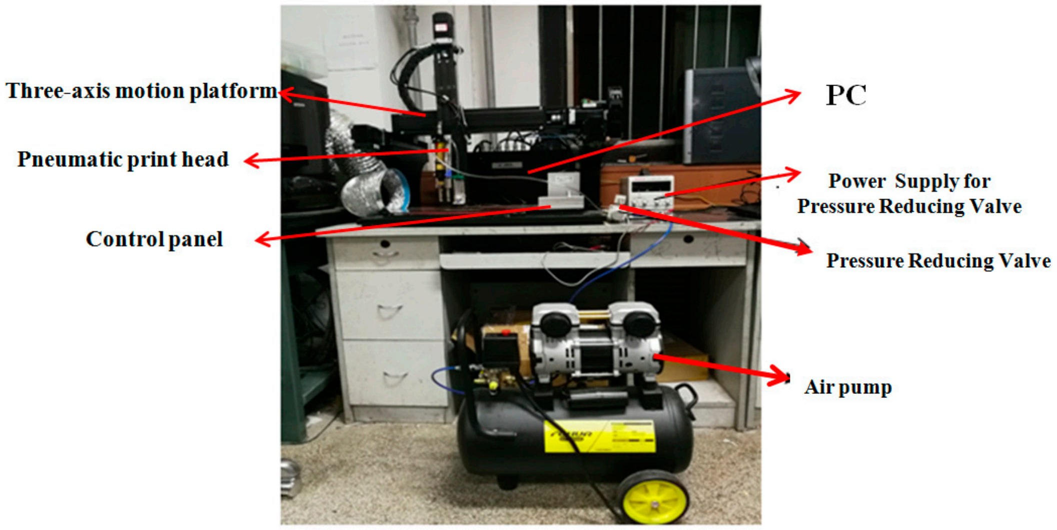

Figure S1. Experimental setup for the direct writing process.

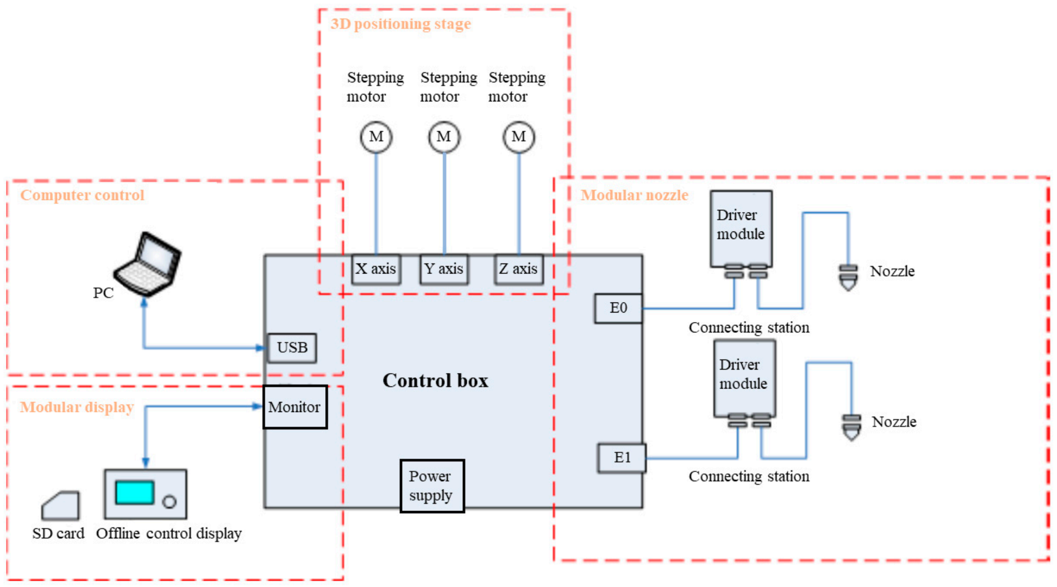

Figure S2. Composition of 3D printing system.

The ink extrusion system consisted of an air pump (FH-30L, Fuhu, P. R. China), a pressure reducing valve (IR2020-02B, SMC, Japan), its power supply (direct current (DC) supply, MS305D, Maisheng, P. R. China), and syringes.

The actuating performance of the PVC gel artificial muscle was measured by a test system (Figure S3, Supporting Information). The displacement of the artificial muscle with a laser sensor (LK-G500, KEYENCE, Japan) was measured using a signal generator (DG4062, RIGOL, P. R. China) and voltage amplifier (MODEL 20/20C, TREK, USA) to apply voltage to the electrode layers of the artificial muscle

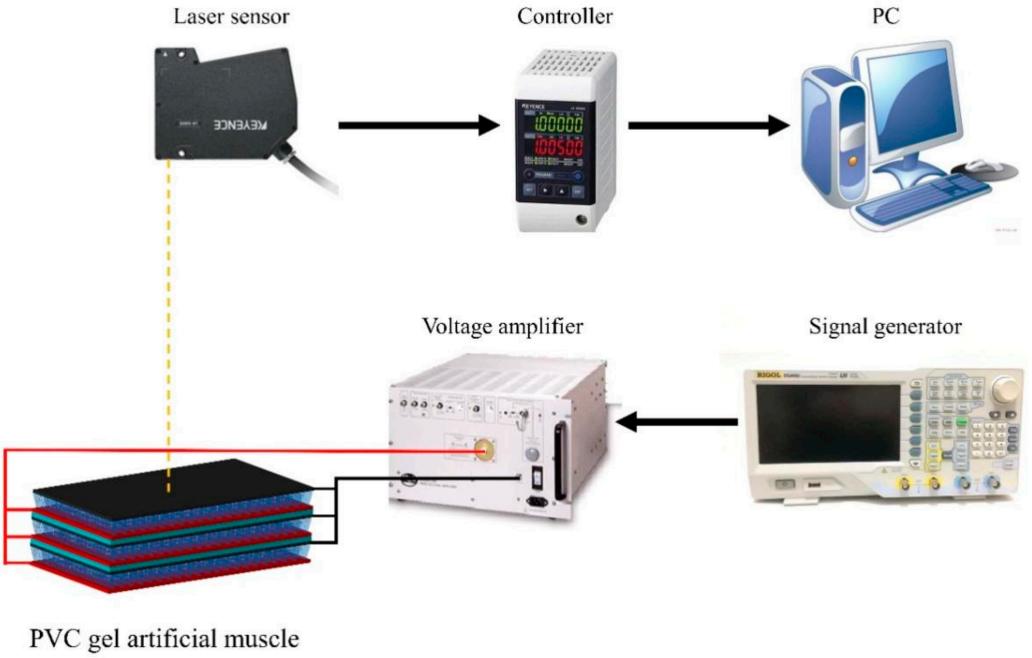

Figure S3. Schematic illustration of the system used to test the PVC gel artificial muscle actuating performance.
